# Reciprocal mediation of immuno-inflammation and insulin resistance in the risk of chronic kidney disease among patients with coronary artery disease: insights from neutrophil-to-lymphocyte ratio and triglyceride-glucose index

**DOI:** 10.3389/fimmu.2026.1937248

**Published:** 2026-09-09

**Authors:** Xiaohong Chen, Min Wang, Bitao Wu, Wenqiang Jiang, Qiang Yang, Jie Tang, Pei Yang, Yuwei Yang

**Affiliations:** 1 Mianyang Central Hospital, School of Medicine, University of Electronic Science and Technology of China, Mianyang, China; 2 NHC Key Laboratory of Nuclear Technology Medical Transformation (Mianyang Central Hospital), Mianyang, China

**Keywords:** chronic kidney disease, coronary artery disease, immuno-inflammation, insulin resistance, mediation, threshold effect

## Abstract

**Objective:**

The neutrophil-to-lymphocyte ratio (NLR), platelet-to-lymphocyte ratio (PLR), and triglyceride-glucose (TyG) index are used to evaluate the risk of coronary artery disease (CAD) or chronic kidney disease (CKD). However, there are limited reports on their role in CKD risk assessment among CAD patients. This study focuses on their linear/nonlinear correlations, threshold effects, and mediating effects with CKD risk strata (classified by KDIGO) among CAD patients to explore their interrelationships.

**Methods:**

This cross-sectional study enrolled 1,091 patients with chronic coronary syndrome (CCS), 166 patients with acute coronary syndrome (ACS), and 431 healthy controls (HC). Their complete blood count indices and glucose/lipid metabolism parameters were used to calculate the NLR/PLR (reflecting immuno-inflammation) and TyG index (reflecting insulin resistance), and their kidney biomarkers were used to evaluate the CKD risk strata based on KDIGO classification.

**Results:**

No significant differences were found between acute and chronic CAD patients in any observational indicators except urea (all P>0.05). As CKD risk strata increased, NLR (z=17.813, P<0.001), PLR (z=8.699, P<0.001), TyG (z=11.213, P<0.001), and insulin resistance (γ=0.471, P<0.001) rose progressively. After adjusting for sex, age, and disease, the NLR and PLR showed linear correlations with potential CKD (P for nonlinear=0.094 and 0.152), with no threshold effects (P for likelihood=0.113 and 0.890); in contrast, TyG showed a nonlinear correlation (P for nonlinear=0.003; Reference threshold=8.62) and a threshold-dependent effect (P for likelihood test<0.001; Inflection point=8.67). Among the three indicators, only the NLR and TyG significantly predicted all four CKD risk strata (NLR OR = 2.30, 2.34, 2.70, and 2.73; TyG OR = 3.80, 3.27, 3.48, and 11.98; all P<0.01). Finally, the NLR and TyG demonstrated a reciprocal mediating role in potential CKD, with their effects strengthening as risk strata increased (NLR Mediation=14.52%~30.16%; TyG Mediation=6.54%~27.07%; all P<0.001).

**Conclusion:**

The NLR and TyG index may serve as potential inflammation-metabolism markers for predicting potential CKD in CAD patients. The reciprocal mediation of NLR and TyG index across CKD risk strata suggests that immuno-inflammation and insulin resistance are overlapping correlates of higher CKD likelihood, rather than fully independent predictors. Combining both indices may improve early identification of high-risk CKD patients and enable early intervention.

## Introduction

Coronary artery disease (CAD) is a chronic cardiovascular condition caused by atherosclerotic narrowing or blockage of the coronary arteries, and remains a leading cause of global morbidity and mortality (1, 2). CAD typically progresses from early atherosclerosis to chronic coronary syndrome (CCS), then to acute coronary syndrome (ACS), and finally to heart failure (3). The term “chronic coronary syndrome” was introduced by the European Society of Cardiology (ESC) in 2019 (4), replacing “stable coronary artery disease” to better reflect disease evolution. The CCS encompasses five subtypes: stable angina, ischemic cardiomyopathy, asymptomatic CAD, stable post-ACS or post-revascularization, and angina suspected to be due to vasospasm or microvascular disease. The ACS encompasses a spectrum of clinical conditions caused by acute myocardial ischemia, including ST-segment elevation myocardial infarction, non-ST-segment elevation myocardial infarction, and unstable angina (5, 6).

Chronic kidney disease (CKD) is an important risk factor for CAD progression, and vice versa (7, 8). It has been documented that the prevalence of CKD in the general population ranges from 6% to 12% (9); however, among patients with CVD, it is at least fivefold higher (8). Consequently, early screening for CKD is essential in patients with CAD. A comprehensive assessment is proposed to integrate both estimated glomerular filtration rate (eGFR) and urine albumin-to-creatinine ratio (ACR), as reliance on eGFR alone would miss approximately 50% of CKD cases (7). Oxidative stress and chronic inflammation are shared pathophysiological pathways of CAD and CKD (10). Their interconnected processes contribute to endothelial dysfunction and atherosclerosis, thereby promoting vascular injury, atherosclerotic plaque formation, increased arterial stiffness, and microalbuminuria, even progressive cardiorenal damage (11). Meanwhile, hypertension, dyslipidemia, obesity, hyperuricemia, and diabetes mellitus (DM) are also shared risk factors for CAD and CKD, collectively establishing the pathogenic network (12, 13). Therefore, the progression of CAD and CKD is mechanistically linked to dysregulated immune-inflammatory responses and underlying metabolic disturbances.

Recent evidence suggests that immuno-inflammatory biomarkers serve as accessible, cost-effective biomarkers for evaluating the presence and severity of CAD and CKD, exemplified by the neutrophil-to-lymphocyte ratio (NLR) and platelet-to-lymphocyte ratio (PLR) (14–16). NLR reflects systemic immune-inflammatory activation and integrates innate (neutrophil) and adaptive (lymphocyte) immunity, offering a practical indicator of immune status in CAD. Likewise, PLR, which is derived from platelet and lymphocyte counts, provides clinically meaningful insights into inflammatory activity and disease burden in CAD patients, reflecting coordinated alterations in thrombosis-inflammation pathways. Notably, insulin resistance status can substantially affect both the NLR and PLR. Insulin resistance is strongly correlated with β-cell dysfunction, fasting hyperglycemia, and poor glycemic control (17, 18). As a result, it can not only predispose patients to various metabolic disorders (including hyperglycemia, dyslipidemia, and hypertension), but also result in elevated NLR and PLR levels, thus worsening CVD and CKD outcomes (19, 20).

Currently, no clinically feasible, widely applicable method exists to precisely quantify insulin resistance. While the euglycemic-hyperinsulinemic clamp and the intravenous glucose tolerance test are regarded as reference standards, their clinical use is limited by complexity, resource intensity, and invasiveness (21). In routine practice, the Homeostatic Model Assessment of Insulin Resistance (HOMA-IR) is the most commonly used surrogate. However, its validity is markedly reduced in patients on exogenous insulin or with advanced β-cell failure, such as in late-stage type 1 or type 2 diabetes (22). The triglyceride-glucose (TyG) index, first introduced in 2008 (23), has been proven to be superior to HOMA-IR in some studies regardless of whether with or without diabetes and insulin therapy (24). Recent evidence also shows the TyG index independently predicts outcomes in CAD and CKD (25, 26), supporting its use for cardiovascular risk stratification and early kidney dysfunction detection.

Overall, immuno-inflammation and insulin resistance synergistically cause kidney injury via multiple immunological pathways: 1) Activated neutrophils release reactive oxygen species, matrix metalloproteinases, and neutrophil extracellular traps that directly damage glomerular endothelial and mesangial cells, promoting glomerulosclerosis and tubulointerstitial fibrosis; 2) Relative lymphocytopenia weakens the tissue-protective function of regulatory T cells, impairing immune surveillance; 3) insulin resistance metabolically activates the polyol pathway, advanced glycation end product formation, and protein kinase C signaling through β-cell dysfunction and persistent hyperglycemia, amplifying oxidative stress and NF-κB-driven pro-inflammatory cytokine release; 4) insulin resistance-induced dyslipidemia and hyperuricemia worsen endothelial dysfunction and renal microvascular damage, increase microalbuminuria, and ultimately accelerate progressive kidney function loss. These immunological pathways form a positive feedback loop: immuno-inflammation ↔ insulin resistance. A nationwide cohort study indicated the co-exposure effect and reciprocal mediating effect of the TyG index and high-sensitivity C-reactive protein on CAD (27). However, there are limited reports on the TyG index as a mediator between immune-inflammatory activation and CKD risk in CAD patients. To address this, this study used the TyG index as a validated insulin resistance surrogate and NLR/PLR as established inflammatory biomarkers to assess their combined and sequential roles in CKD risk among CAD patients.

## Methods

### Participants

This cross-sectional study sequentially enrolled 1091 CCS outpatients (711 with stable angina, 327 with ischemic cardiomyopathy, and 53 with silent CAD) and 166 ACS inpatients (105 with unstable angina, 42 with non-ST-segment elevation myocardial infarction, and 19 with ST-segment elevation myocardial infarction). These patients were either visited or admitted to the Department of Cardiovascular Medicine at Mianyang Central Hospital, School of Medicine, University of Electronic Science and Technology of China between January and December 2025. Laboratory data on kidney biomarkers, glucose/lipid metabolism, and complete blood count within 3 days after admission were collected, along with a history of chronic conditions.

Inclusion criteria: 1) Aged 35 years or older; 2) Diagnosis of CSS or ACS per ESC guidelines (4, 28); 3) Asymptomatic CAD was defined as ≥70% diameter stenosis in any coronary artery, confirmed by coronary or CT angiography; 4) Symptomatic CAD pertained to patients presenting with angina (clinically diagnosed, with or without confirmatory testing) or a positive stress test; 5) Treated CAD referred to patients who had undergone angioplasty, stenting, or revascularization; 6) Measurement of serum/urinary kidney biomarkers, serum glucose/lipid profile, and complete blood count within 3 days of admission, all data could be collected completely.

Exclusion criteria: 1) Any comorbidities that interfere with NLR, TyG and CKD assessment, such as severe infection, pulmonary failure, primary nephropathy, autoimmune disease, thyroid disorder, or malignancy; (4) Use within the past week of medications affecting lipid metabolism, glucose control, or systemic inflammation, such as corticosteroids, immunosuppressants, or lipid-lowering drugs; 5) Any other conditions that alter metabolism or inflammation, such as recent surgery, alcohol abuse, or substance abuse; 6) Incomplete data for NLR, TyG, or CKD assessment.

Meanwhile, this study enrolled 431 healthy individuals matched by sex and age. All had normal liver and kidney function and negative blood and urine routine tests. Their kidney biomarkers, glucose/lipid metabolism parameters, and complete blood count indices were systematically measured.

### Sample collection and quantitative analysis

Two fasting morning blood samples were collected from all participants: one in an EDTA-containing tube for complete blood count and glycated hemoglobin A1c (HbA1c) measurement, and another in a serum separator tube with clot activator and gel for the assessment of fasting plasma glucose (Glu), lipid profile, and kidney biomarkers. Additionally, midstream urine samples were collected within two hours before or after blood collection to measure the urinary albumin-to-creatinine ratio (ACR).

Leukocyte differentials and platelet counts were measured via sheath-flow flow cytometry on a Sysmex XN-9000 analyzer (Sysmex, Japan) to calculate the neutrophil-to-lymphocyte ratio (NLR) and platelet-to-lymphocyte ratio (PLR). HbA1c was measured via column chromatography on a Variant II TURBO analyzer (Bio-Rad, USA). Glu, lipid profile, and kidney biomarkers were measured on an LST008 biochemical autoanalyzer (HITACHI, Japan) using Maccura reagents (Sichuan, China). This study encompassed the following biomarkers and methodologies: Glu, hexokinase method; urea (Ure), urease method; creatinine (Cre), sarcosine oxidase method; uric acid (UA), uricase coupled peroxidase method; triglyceride (TG), glycerol phosphate oxidase method; total cholesterol (T-Chol), cholesterol oxidase method; high-density lipoprotein cholesterol (HDL-Chol) and low-density lipoprotein cholesterol (LDL-Chol), immunoturbidimetric method. Estimated glomerular filtration rate (eGFR) was calculated using the corrected M-MDRD equation (29): eGFR = 234.96 × Cr^-0.926^ × Age^-0.280^ × 0.828(if female), with Cr in mg/dL. Triglyceride-Glucose (TyG) index was calculated using the formula (30): TyG = ln [(TG × Glu)/2], with TG and Glu in mg/dL. According to established clinical criteria, insulin resistance was defined as a TyG index greater than 8.81 in males and greater than 8.73 in females (31).

Urinary albumin was measured by modified immunoturbidimetry and urinary creatinine by the sarcosine oxidase method, both on a BioSystems A25 analyzer (Spain) using Biostec reagents (Chongqing, China). ACR was calculated using the following formula: ACR (mg/g) = urinary albumin (mg/L)/urinary creatinine (g/L).

### CKD risk strata based on KDIGO classification

All 1,091 CAD patients were classified into four CKD risk strata groups per KDIGO 2024 guidelines (32): 1) Mild CKD (n=616), wherein ACR<30mg/g and eGFR ≤60ml/min/1.73m^2^; 2) Moderate CKD (n=265), wherein ACR = 30~300mg/g and eGFR ≤60ml/min/1.73m^2^, or ACR <30mg/g and eGFR=45~59ml/min/1.73m^2^; 3) Severe CKD (n=105), wherein ACR>300mg/g and eGFR ≤ 60ml/min/1.73m^2^, or ACR = 30-300mg/g and eGFR=45~59 ml/min/1.73m^2^, or ACR <30mg/g and eGFR=30~44ml/min/1.73m^2^; 4) Extremely severe CKD (n=105), wherein ACR>300mg/g and eGFR=45~59ml/min/1.73m^2^, or ACR ≥30mg/g and eGFR=30~44ml/min/1.73m^2^, or eGFR <30ml/min/1.73m^2^ regardless of ACR levels. As confirmatory testing every 1–2 months over 3–6 months was not performed, this study defined a single test of ACR ≥30mg/g or eGFR <60mL/min/1.73m^2^ as potential CKD.

### Statistical analysis

Data analysis was performed via MedCalc software v22.021 (MedCalc Software Ltd, Ostend, West Flanders, Belgium), SPSS software v22.0 (SPSS, Armonk, NY, USA), or Zstats Online Statistical Platform (https://www.medsta.cn/software). Continuous data are presented as the median (25th percentile, 75th percentile) and count data as frequency (percentage). Multiple-group differences were analyzed via the Kruskal-Wallis test (for continuous data) or the Chi-square test (for count data), then *post-hoc* analyses were performed with the Bonferroni correction; statistical significance was determined using adjusted p-value (Padj). Changing trend was analyzed via the Jonckheere-Terpstra test (for continuous data) or the Gamma consistency test (for count data). Restricted cubic spline (RCS) and threshold effect analyses were used to analyze the linear/nonlinear correlations and the dose-response relationships between NLR/PLR/TyG and potential CKD, respectively. Correlation magnitudes of CKD risk strata with TyG and immuno-inflammatory biomarkers (NLR and PLR) were determined via multinomial logistic regression analysis adjusted for confounding factors, and the odds ratio (OR) and its 95% confidence interval (CI) were used for measurement. In sensitivity analyses, the Bonferroni correction was applied for subgroup analyses; statistical significance was determined using adjusted p-value (Padj). The significance level was set at α=0.05.

## Results

### Demographic characteristics and laboratory data of the participants

This study achieved well-balanced sex and age distributions across HC, CCS, and ACS groups (all P>0.05; **Table 1**). Compared with the HC group, both CCS and ACS groups showed significant differences in kidney biomarkers, glucose/lipid metabolism, immunoinflammatory markers, and insulin resistance indices (all Padj<0.05). However, the CCS and ACS groups did not differ significantly for most parameters or chronic conditions, except urea (z=2.778, Padj=0.016) and hyperlipidemia (χ2 = 7.118, P = 0.008), both higher in the ACS group. Thus, the CCS and ACS groups exhibited similar glucose/lipid metabolism, immuno-inflammation, insulin resistance, and kidney function, so separate analyses were unwarranted.

**Table 1:** Comparison of demographic characteristics and laboratory data among three groups.

Parameters	HC (n=431)	CCS (n=1091)	ACS (n=166)	χ2	P
Male [n (%)] *	230 (53.36)	554 (50.78)	88 (53.01)	0.962	0.618
Age (years)	71 (60, 78)	72 (62, 81)	73 (60, 79)	3.672	0.159
ACR (mg/g)	7.15 (4.85, 11.56)	11.96 (4.98, 34.74) ^▲^	6.72 (3.48, 14.20) ^▲^	71.702	<0.001
eGFR (ml/min/1.73m^2^)	91.1 (80.7, 99.6)	76.0 (56.8, 90.8) ^▲^	73.3 (61.2, 90.0) ^▲^	174.213	<0.001
Ure (mmol/L)	5.38 (4.39, 6.37)	6.06 (4.96, 7.97) ^▲^	5.41 (4.68, 7.00) ^▲Δ^	72.862	<0.001
Cre (μmol/L)	73.0 (63.9, 84.5)	79.2 (65.0, 98.9) ^▲^	81.0 (70.8, 95.9) ^▲^	38.821	<0.001
UA (μmol/L)	303 (257, 363)	321 (258, 399) ^▲^	323 (267, 400) ^▲^	16.336	<0.001
HbA1c (%)	5.7 (5.40, 6.00)	6.1 (5.6, 6.9) ^▲^	6.2 (5.7, 6.8) ^▲^	177.977	<0.001
Glu (mmol/L)	4.85 (4.51, 5.25)	5.71 (4.93, 7.67) ^▲^	5.96 (5.13, 7.46) ^▲^	274.650	<0.001
TG (mmol/L)	1.16 (0.87, 1.44)	1.26 (0.88, 1.83) ^▲^	1.22 (1.01, 1.97) ^▲^	22.522	<0.001
TC (mmol/L)	3.47 (2.96, 3.91)	3.77 (3.02, 4.67) ^▲^	3.89 (3.16, 4.85) ^▲^	87.050	<0.001
HDL-C (mmol/L)	1.30 (1.09, 1.59)	1.25 (1.04, 1.50) ^▲^	1.25 (1.06, 1.50) ^▲^	9.999	0.007
LDL-C (mmol/L)	1.68 (1.22, 2.06)	2.04 (1.43, 2.86) ^▲^	2.09 (1.41, 2.86) ^▲^	84.601	<0.001
TyG	8.43 (8.13, 8.67)	8.74 (8.25, 9.19) ^▲^	8.77 (8.33, 9.24) ^▲^	124.961	<0.001
NLR	1.76 (1.37, 2.31)	2.75 (1.92, 3.91) ^▲^	2.41 (1.95, 3.29) ^▲^	231.411	<0.001
PLR	98.4 (79.1, 128.7)	119.1 (90.2, 166.2) ^▲^	111.9 (93.3, 180.0) ^▲^	58.340	<0.001
IR [n (%)] *	65 (15.08)	522 (47.85) ^▲^	88 (53.01) ^▲^	151.221	<0.001
Hypertension [n (%)] *	NS	651 (59.67)	96 (57.83)	0.202	0.653
Hyperlipemia [n (%)] *	NS	352 (32.26)	71 (42.77) ^Δ^	7.118	0.008
Hyperuricemia [n (%)] *	NS	268 (24.56)	50 (30.12)	2.351	0.125
DM [n (%)] *	NS	382 (35.01)	63 (37.95)	0.543	0.461

*Using the chi-square test; otherwise, the Kruskal-Wallis test was used. ^▲^vs. HC, Padj<0.05; ^Δ^vs. CCS, P<0.05. HC, health controls; CCS, chronic coronary syndrome; ACS, acute coronary syndrome; ACR, albumin-to-creatinine ratio; eGFR, estimated glomerular filtration rate; Ure, urea; Cre, creatinine; UA, uric acid; HbA1c, glycated hemoglobin A1c; Glu, glucose; TG, triglyceride; TC, total cholesterol; HDL-C, high-density lipoprotein cholesterol; LDL-C, low-density lipoprotein cholesterol; TyG, triglyceride-glucose index; NLR, neutrophil to lymphocyte ratio; PLR, platelet to lymphocyte ratio; DM, diabetes mellitus; IR, insulin resistance; NS, no statistics.

### Increased immuno-inflammation and insulin resistance with advancing CKD risk

**Figure 1:**
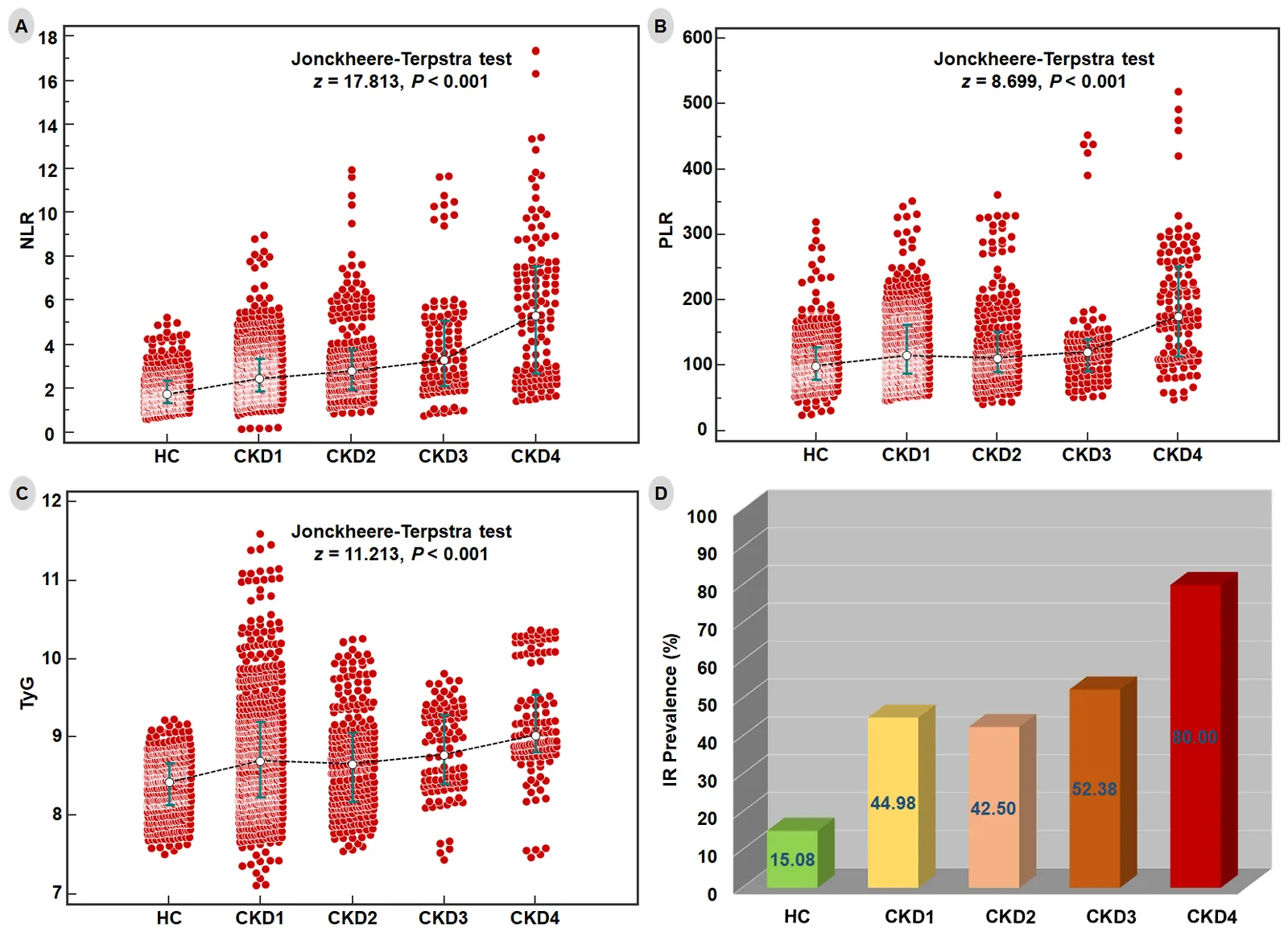
Trends of NLR, PLR, TyG index, and insulin resistance with CKD risk strata. **(A)** NLR; **(B)** PLR; **(C)** TyG index; **(D)** IR Prevalence. Red dots denote individual data; white dots represent the median, and the I-shaped markers indicate the 25th and 75th percentiles, respectively. As risk strata increased, the NLR, PLR, TyG index, and insulin resistance status exhibited significant increasing trends. HC, health controls; CKD1 to CKD4 represent mild, moderate, severe, and extremely severe CKD risks, respectively; TyG, triglyceride-glucose index; NLR, neutrophil to lymphocyte ratio; PLR, platelet to lymphocyte ratio; IR, insulin resistance.

The Jonckheere-Terpstra test revealed a significantly gradual increase in the NLR (z=17.813, P<0.001), PLR (z=8.699, P<0.001), and TyG index (z=11.213, P<0.001) across CKD risk strata (**Figures 1A–C**). Participants’ insulin resistance status demonstrated a high gamma consistency with CKD risk strata (γ=0.471, 95%CI=0.410 to 0.535, statistic T = 13.847, P<0.001) (**Figure 1D**).

### Threshold effect of insulin resistance on the potential occurrence of CKD

**Figure 2:**
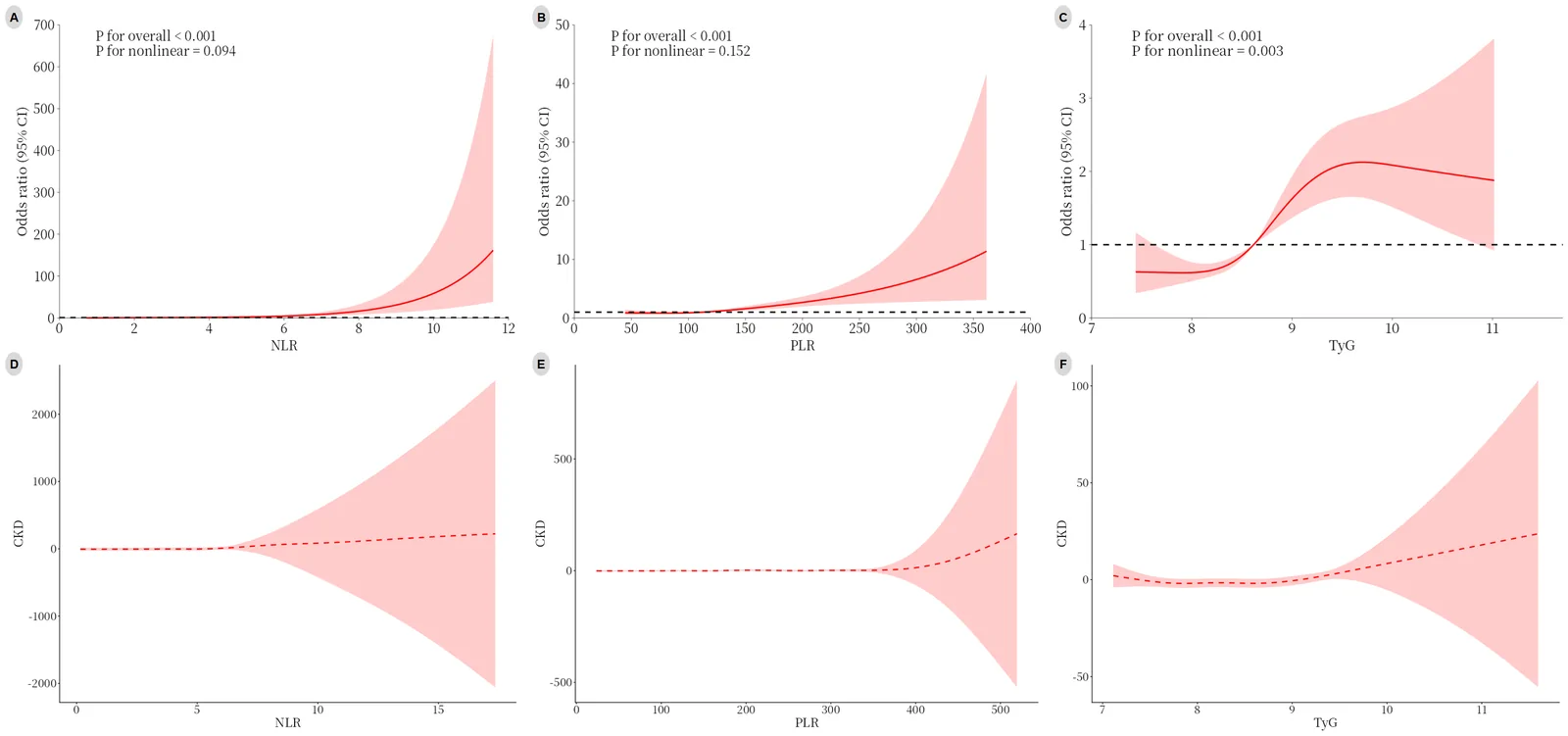
Restricted cubic spline plots and dose-response curves of NLR, PLR, and TyG with potential CKD. Adjusted by sex, age, and disease. Restricted cubic spline plots of: **(A)** Neutrophil-to-lymphocyte ratio (NLR); **(B)** Platelet-to-lymphocyte ratio (PLR); **(C)** Triglyceride-glucose index (TyG). Dose-response curves of: **(D)** NLR; **(E)** PLR; **(F)** TyG. The RCS analysis revealed linear correlations between NLR/PLR and potential CKD, but a nonlinear correlation for TyG. The dose-response curves illustrated a threshold effect of TyG on CKD, absent for NLR and PLR. CI, confidence interval.

The RCS analyses revealed linear correlations of NLR and PLR with potential CKD (both P for overall<0.001, P for nonlinear=0.094 and 0.152), with estimated reference thresholds of 2.38 and 112.6, respectively (**Figures 2A, B**). In contrast, TyG exhibited a nonlinear correlation with potential CKD (P for overall<0.001, P for nonlinear=0.003), with an estimated reference threshold of 8.62 (**Figure 2C**). Subsequent threshold effect analyses revealed no significant threshold effects in the associations between NLR/PLR and potential CKD (P for likelihood test=0.113 and 0.890), with estimated inflection points at 2.31 and 59.3, respectively (**Figures 2D, E**; **Table 2**). However, TyG exhibited a significant threshold effect on potential CKD (P for likelihood test<0.001), with an estimated inflection point of 8.67 (**Figure 2F**, **Table 2**). In these two analyses, the node values for NLR and TyG were comparable, whereas those for PLR exhibited an apparent disparity (112.6 vs. 59.3).

**Table 2:** Threshold effect analyses of NLR, PLR, and TyG on potential CKD.

Outcome	NLR		PLR		TyG	
	Effect (95% CI)	*P*	Effect (95% CI)	*P*	Effect (95% CI)	*P*
Model 1	2.438 (2.112, 2.814)	<0.001	1.009 (1.006, 1.011)	<0.001	3.368 (2.694, 4.212)	<0.001
Model 2						
Inflection point	2.31		59.3		8.67	
< Inflection point	3.255 (2.226, 4.758)	<0.001	1.077 (1.000, 1.160)	0.049	0.606 (0.373, 0.984)	0.043
≥ Inflection point	1.978 (1.534, 2.550)	<0.001	1.009 (1.007, 1.012)	<0.001	132.007 (35.178, 495.366)	<0.001
P for likelihood test		0.113		0.890		<0.001

Modes 1 and 2 were fitted using standard linear regression and two-piecewise linear regressions respectively, adjusting by sex, age, and disease. Among the immune-inflammatory and insulin resistance biomarkers evaluated, only the TyG index demonstrated a statistically significant threshold effect on potential CKD. NLR, neutrophil to lymphocyte ratio; PLR, platelet to lymphocyte ratio; TyG, triglyceride-glucose index; CI, confidence interval.

### Associations of NLR, PLR, and TyG with CKD risk strata

Referenced to HC and adjusted by sex, age, and disease, the multinomial logistic regression analysis revealed that the NLR and TyG index demonstrated significant predictive value across various CKD risk strata [Mild: OR(95%CI)=2.30(1.78, 3.19) and 3.80(2.40, 6.73); Moderate: OR(95%CI)=2.34(1.76, 3.56) and 3.27(1.74, 6.42); Severe: OR(95%CI)=2.70(1.96, 4.75) and 3.48(1.40, 8.13); Extremely severe: OR(95%CI)=2.73(1.99, 5.26) and 11.98(6.11, 30.50); all P<0.05] (**Table 3**). The PLR did not demonstrate significant predictive value for potential CKD (all P>0.05).

**Table 3:** The magnitude and odds ratios associated with NLR, PLR, and TyG in predicting potential CKD.

CKD risk	Beta	Bootstrap (n=1000)				OR (95% CI)	Wald	P
		SE	Bias	Lower	Upper			
Mild								
NLR	0.831	0.023	0.150	0.579	1.160	2.30 (1.78, 3.19)	32.203	0.001
PLR	-0.031	-0.002	0.036	-0.103	0.036	0.97 (0.90, 1.04)	0.993	0.390
TyG	1.335	0.022	0.260	0.877	1.906	3.80 (2.40, 6.73)	25.718	0.001
Moderate								
NLR	0.851	0.047	0.183	0.566	1.269	2.34 (1.76, 3.56)	27.886	0.001
PLR	-0.043	-0.009	0.048	-0.152	0.037	0.96 (0.86, 1.04)	1.271	0.348
TyG	1.184	0.013	0.336	0.551	1.859	3.27 (1.74, 6.42)	12.416	0.001
Severe								
NLR	0.992	0.083	0.214	0.674	1.558	2.70 (1.96, 4.75)	32.287	0.001
PLR	-0.090	-0.028	0.079	-0.271	0.031	0.91 (0.76, 1.03)	2.630	0.212
TyG	1.246	-0.021	0.450	0.337	2.095	3.48 (1.40, 8.13)	6.535	0.004
Extremely severe								
NLR	1.005	0.101	0.243	0.687	1.659	2.73 (1.99, 5.26)	34.848	0.001
PLR	0.027	-0.005	0.070	-0.127	0.159	1.03 (0.88, 1.17)	0.345	0.684
TyG	2.483	0.069	0.411	1.810	3.418	11.98 (6.11, 30.50)	32.906	0.001

Referenced to HC and adjusted by sex, age, and disease. The NLR and TyG index demonstrated significant predictive value for potential CKD, whereas the PLR did not. NLR, neutrophil to lymphocyte ratio; PLR, platelet to lymphocyte ratio; TyG, triglyceride-glucose index; CKD, chronic kidney disease; SE, standard error; OR, odds ratio.

Model fitting:-2 Log Likelihood = 3744.505, χ2 = 890.284, P < 0.001, Cox and Snell = 0.410, Nagelkerke = 0.438.

### Mediating role of TyG in NLR-induced renal dysfunction

**Figure 3:**
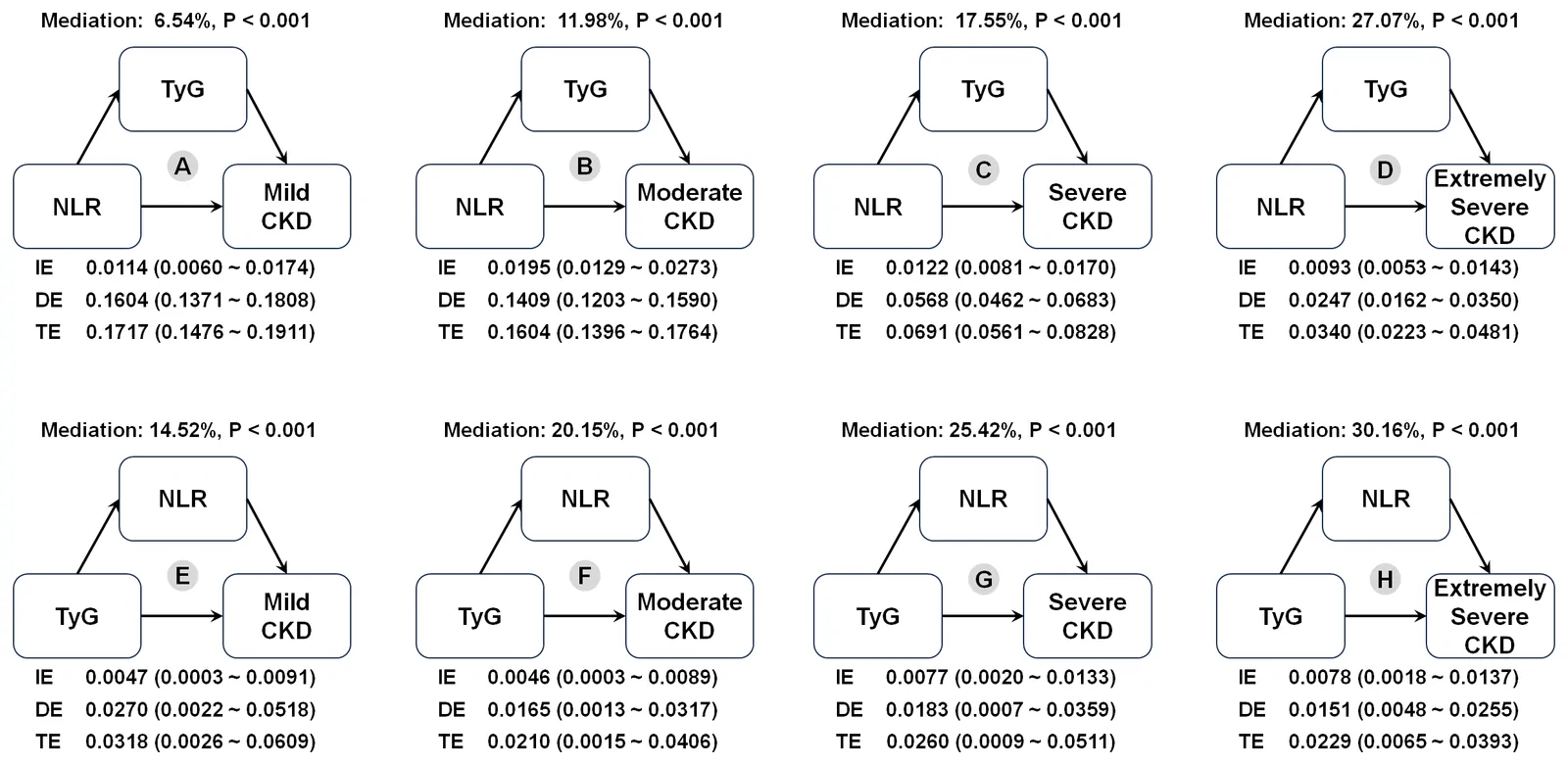
Mediating effects between NLR and various CKD risks. Referenced to HC and adjusted by sex, age, and disease. TyG as a mediating variable across four risks: **(A)** mild CKD; **(B)** moderate CKD; **(C)** severe CKD; and **(D)** extremely severe CKD. NLR as a mediating variable across four risks: **(E)** mild CKD; **(F)** moderate CKD; **(G)** severe CKD; and **(H)** extremely severe CKD. The NLR and TyG index reciprocally mediated potential CKD, with effects strengthening as risk strata increased. NLR, neutrophil-to-lymphocyte ratio; TyG, triglyceride-glucose index; CKD, chronic kidney disease; IE, indirect effect; DE, direct effect; TE, total effect.

Referenced to HC and adjusted by sex, age, and disease, the mediation analysis indicated that TyG exerted mediating effects in the NLR-CKD association across four CKD risk strata (from mild to extremely severe CKD: Mediation=6.54%, 11.98%, 17.55%, and 27.07, all P<0.001) (**Figures 3A–D**). Meanwhile, NLR also exerted mediating effects in the TyG-CKD association across four CKD risk strata (from mild to extremely severe CKD: Mediation=14.52%, 20.15%, 25.42%, and 30.16, all P<0.001) (**Figures 3E–H**).

### Subgroup sensitivity analysis

Subgroup sensitivity analysis revealed consistent RCS correlation and threshold effect patterns of the TyG index, NLR, and PLR in CCS and ACS patients: the TyG index had a nonlinear correlation and threshold-dependent effect with potential CKD, while NLR and PLR had linear correlation and threshold-free effects (**Supplementary Figure 1**; **Supplementary Table 1**). Furthermore, reciprocal mediation of TyG index and NLR across CKD risk strata was observed in both CCS and ACS patients, with the effect magnitude increasing alongside rising CKD risk (**Supplementary Table 2**). These findings were consistent with the results of the overall analysis, with comparable estimated reference thresholds, inflection points, and mediation magnitude.

## Discussion

Accelerating experimental evidence suggests that NLR, PLR, and TyG index are closely associated with CAD and CKD occurrence and progression (25, 33–35). Nevertheless, there are limited reports on the relationship between these three indices and CKD risk in CAD patients. This study not only reconfirms that NLR and TyG index are independent predictors of CKD risk in CAD patients but also provides more in-depth experimental perspectives on the association between immuno-inflammation and metabolic dysregulation. Specifically, the following innovative findings were observed: 1) Both CCS and ACS were characterized by consistent inflammation and insulin resistance, which are associated with the increased risk of CKD; 2) NLR demonstrated a linear, threshold-free association with CKD risk, highlighting a central role of immune inflammation in progressive CKD risk; 3) TyG index exhibited a nonlinear, threshold-dependent pattern with a risk accelerator effect beyond a identified threshold, underscoring insulin resistance-related metabolic dysregulation as a crucial predictor of higher CKD likelihood; 4) NLR and TyG index demonstrated a reciprocal mediating role in CKD risk, with their effects strengthening as risk increased, suggesting reciprocal activation and synergistic amplification of immune-inflammation and insulin resistance; 4) PLR is linearly correlated with CKD risk but lacks independent predictive value and has limited clinical relevance.

Growing interest in NLR and PLR lies in their practical advantages in clinical utility, including cost-effectiveness, straightforward operation, fast results, and ease of access. However, evidence linking NLR/PLR to clinically meaningful outcomes in CKD patients remains inconsistent across studies. In this study, although both NLR and PLR exhibited an increasing trend across CKD risk strata among CAD patients, only NLR demonstrated independent predictive ability, whereas PLR lacked independent predictive value. NLR and PLR capture fundamentally distinct pathophysiological processes, and this biological divergence accounts for their contrasting predictive performance in this study. CKD is characterized by low-grade chronic inflammation and immune dysregulation; NLR, as a well-established biomarker of systemic immune-inflammatory activation, is therefore inherently aligned with CKD pathogenesis. Consistent with this biological rationale, NLR has been consistently associated with CKD progression and a broad spectrum of adverse outcomes, including poor nutritional status, mental health disorders, all-cause mortality, cardiovascular events, cardiovascular mortality, and adverse renal outcomes (33, 36–38). In contrast, PLR, as an established biomarker of thrombosis-inflammatory activation, primarily reflects a prothrombotic state. Although CKD carries thrombosis risk (a bleeding-thrombosis coexistence phenomenon) from coagulation factor deficiency, hyperfibrinolysis, and drug effects (39, 40), thrombosis only acts as a secondary complication rather than the primary cause of CKD, while it is the core pathogenic driver of CAD. In the CAD population, the baseline prothrombotic state is already so pervasive and pronounced that PLR’s discriminative capacity for CKD risk strata is effectively diluted. This interpretation is consistent with prior evidence that PLR holds predictive value only in patients with advanced CKD (33). Further underscoring PLR’s limited clinical relevance, a distinct discrepancy was observed between the PLR nodes identified by RCS analysis (reference threshold) and those obtained from threshold effect analysis (inflection point). Subsequent findings regarding the distinct discrepancy between PLR nodes from RCS analysis (reference threshold) and threshold effect analysis (inflection point) further highlight its limited clinical relevance, as this discrepancy may suggest that the association between PLR and CKD risk is unstable or severely confounded in the CAD population.

Our study demonstrates that the NLR and TyG index are important inflammation-metabolism biomarkers for CKD risk strata in CAD patients. After adjusting for sex, age, and disease, a linear correlation was observed between NLR and CKD risk strata, with no significant threshold effect detected. A linear association between NLR and kidney injury has been consistently reported in multiple studies on other diseases (25, 41–43); however, evidence regarding potential threshold effects remains scarce. A cross-sectional study found a threshold effect of NLR on reduced eGFR, without significant association below the inflection point (44). NLR reflects chronic cumulative immunoinflammatory burden: a continuous spectrum of immune dysregulation that progressively erodes renal reserve. Its underlying mechanisms are increasingly clear: sustained neutrophilia damages renal parenchyma via releasing reactive oxygen species, matrix metalloproteinases, and neutrophil extracellular traps, while relative lymphopenia indicates impaired adaptive immune surveillance and reduced regulatory T-cell-mediated tissue protection. This dual immune perturbation is not “on-off”, but intensifies gradually with advancing metabolic stress, endothelial dysfunction, and subclinical renal injury, manifesting as a continuous risk gradient rather than a threshold-dependent phenomenon. Our findings suggest that the immuno-inflammation, as reflected by NLR, progressively increases CKD risk strata among CAD patients in a continuous linear manner, with no critical threshold for a sudden risk shift. This conclusion has both biological and clinical implications. Biologically, it reinforces the view that CKD risk in CAD is correlated to a sustained, graded immunoinflammatory process rather than a discrete pathological trigger. Clinically, it suggests inflammation-targeted risk reduction strategies should not be predicated on exceeding a specific NLR cutoff, but instead aim to lower inflammatory burden across the entire continuum, as any reduction can deliver proportional renal benefits.

Strong evidence shows insulin resistance is the key mechanism linking the TyG index to CKD. Insulin resistance can impair endothelial function and accelerates CKD progression through multiple mechanisms: first, it disrupts glucose metabolism, leading to hyperglycemia, inflammation, and oxidative stress (25); second, it increases the production of advanced glycation end products and free radicals, inactivating nitric oxide and impairing endothelium-dependent vasodilation to weaken barrier function (45); third, it activates the mitochondrial electron transport chain, excessively generating reactive oxygen species, thereby further damaging tubular endothelium (46). In this study, the TyG index exhibited a nonlinear, threshold-dependent pattern with a risk accelerator effect, and the thresholds estimated from RCS and threshold-effect analyses were extremely close. Numerous studies on other diseases consistently indicate this nonlinear association and threshold effect between the TyG index and CKD, with thresholds ranging from 7.05 to 8.94; notably, those for cardiovascular diseases tend to be higher (47–50). These findings suggest that insulin resistance exerts an accelerating effect on CKD risk: risk rises gradually below the threshold but surges sharply above it. Notably, the predictive efficacy of the TyG index for extremely severe CKD far exceeds that of NLR, likely due to its nonlinear threshold effect, making it a stronger risk accelerator in advanced CKD. Furthermore, a reciprocal mediating relationship was observed between the NLR and TyG index in predicting CKD risk among CAD patients, and their mediating effect increased progressively with the severity of CKD risk. These findings reveal a positive interplay between inflammation and insulin resistance in increasing CKD risk: they mutually amplify each other’s CKD-promoting effect, jointly raising the likelihood of CKD from mild to severe. Well-designed interventional trials are warranted to rigorously evaluate the efficacy of anti-inflammatory agents in ameliorating insulin resistance and mitigating CKD risk.

Limitations: This cross-sectional study cannot establish causality, and further prospective cohort studies are needed to confirm the mediation pathways. Ignoring the test of another key pathogenic mechanism (oxidative stress pathway) and confounding or influencing factors may lead to an unbalanced conclusion. Future studies should incorporate additional potential confounding variables (including oxidative stress biomarkers, hypertension status, diabetes duration, body mass index, smoking history, and concomitant pharmacotherapy), and may further extend analyses to immune cell subsets, such as neutrophil, monocyte, and lymphocyte subpopulations, as well as key medications. Relying on a single test for CKD risk assessment and a single-center design may introduce outcome bias. Future studies should use a multiple-cohort design with at least 3-month CKD follow-up for external validation and rigorous evaluation.

## Conclusions

Our study identifies the NLR and TyG index as crucial inflammation-metabolism markers for assessing CKD risk in CAD patients. As a biomarker of systemic immuno-inflammation, NLR demonstrates a linear, threshold-free association with CKD risk, whereas the TyG index, as a surrogate indicator of insulin resistance, exhibits a nonlinear threshold-dependent effect, rising sharply beyond a critical value. Both biomarkers can independently predict all CKD stages, and they mutually mediate each other’s effect, with mediation intensifying as CKD risk increases. These findings reveal a pathophysiological loop where immuno-inflammation and insulin resistance reinforce one another to increase the likelihood of CKD.

When aiming to mitigate CKD risk in clinical practice, the following aspects should be taken into consideration: 1) Given the linearly progressive association between NLR and CKD risk, a sustained elevation of inflammatory markers warrants careful monitoring; 2) TyG index crossing its threshold suggests a sharp increase in CKD risk; 3) Combining NLR and TyG is beneficial for enhancing the identification of high-risk CKD patients; 4) Targeted interventions leveraging this reciprocal mediation may yield more effective mitigation of CKD risk.

## Data Availability

The raw data supporting the conclusions of this article will be made available by the authors, without undue reservation.

## Supplementary material

Supplementary Figure 1 Adjusted by sex, age, and disease. Restricted cubic spline plots of NLR, PLR, and TyG in: ABC) CCS subgroup; DEF) ACS subgroup. Dose-response curves of NLR, PLR, and TyG in: GHI) CCS subgroup; JKL) ACS subgroup. Subgroup findings of RCS correlation, threshold effect, and mediation patterns aligned with the overall analysis, with comparable reference thresholds, inflection points, and mediation magnitudes.
