# Supplementary material for: Reciprocal mediation of immuno-inflammation and insulin resistance in the risk of chronic kidney disease among patients with coronary artery disease: insights from neutrophil-to-lymphocyte ratio and triglyceride-glucose index
Source: Front Immunol. 2026 Sep 9;17:1937248. doi: 10.3389/fimmu.2026.1937248 (PMC13597357; doi:10.3389/fimmu.2026.1937248)
Supplement: Supplementary file 1 [file DataSheet1.docx]

**Supplementary Table 1. Subgroup Analyses for Threshold Effect.**

| Outcome | NLR | | PLR | | TyG | |
| --- | --- | --- | --- | --- | --- | --- |
|  | Effect(95%CI) | *Padj* | Effect(95%CI) | *Padj* | Effect(95%CI) | *Padj* |
| CCS |  |  |  |  |  |  |
| Model 1 | 2.428(2.105, 2.800) | <0.001 | 1.009(1.006, 1.011) | <0.001 | 3.450(2.738, 4.346) | <0.001 |
| Model 2 |  |  |  |  |  |  |
| Inflection point | 1.98 |  | 80.82 |  | 8.68 |  |
| < Inflection point | 0.190(0.012, 3.150) | 0.247 | 0.990(0.968, 1.013) | 0.389 | 0.602(0.365, 0.993) | 0.047 |
| ≥ Inflection point | 2.383(2.045, 2.778) | <0.001 | 1.011(1.008, 1.014) | <0.001 | 144.880(36.990, 567.459 | <0.001 |
| P for likelihood test |  | 0.579 |  | 0.062 |  | <0.001 |
| ACS |  |  |  |  |  |  |
| Model 1 | 2.076(1.686, 2.557) | <0.001 | 1.008(1.005, 1.012) | <0.001 | 6.276(4.033, 9.764) | <0.001 |
| Model 2 |  |  |  |  |  |  |
| Inflection point | 2.34 |  | 46.39 |  | 8.84 |  |
| < Inflection point | 1.000(0.812, 1.150) | 0.677 | 1.000(0.991, 1.009) | 0.949 | 1.172(0.584, 2.350) | 0.655 |
| ≥ Inflection point | 1.627(1.311, 2.020) | <0.001 | 1.016(1.008, 1.025) | <0.001 | 97.136(8.890, 191.733) | <0.001 |
| P for likelihood test |  | 0.103 |  | 0.087 |  | <0.001 |

Note: Modes 1 and 2 were fitted using standard linear regression and two-piecewise linear regressions respectively, adjusting by sex, age, and disease. The TyG index, NLR, and PLR exhibited subgroup threshold effect patterns consistent with those observed in the overall analysis, with similar inflection points. NLR, neutrophil to lymphocyte ratio; PLR, platelet to lymphocyte ratio; TyG, triglyceride-glucose index; CI, confidence interval.

**Supplementary Table 2. Subgroup Analyses of Mediation Effect.**

| CKD risk strata | TyG as mediator | | | | | | | NLR as mediator | | | | | |
| --- | --- | --- | --- | --- | --- | --- | --- | --- | --- | --- | --- | --- | --- |
|  | Effect | Estimate | SE | Lower | Upper | Padj | Mediation | Estimate | SE | Lower | Upper | Padj | Mediation |
| CCS patients |  |  |  |  |  |  |  |  |  |  |  |  |  |
| Mild | Indirect | 0.0108 | 0.0028 | 0.0054 | 0.0162 | <0.001 | 6.44 | 0.0356 | 0.0170 | 0.0023 | 0.0688 | <0.001 | 14.57 |
|  | Direct | 0.1529 | 0.0102 | 0.1329 | 0.1729 | <0.001 |  | 0.2045 | 0.0984 | 0.0116 | 0.3974 | <0.001 |  |
|  | Total | 0.1637 | 0.0106 | 0.1429 | 0.1845 | <0.001 |  | 0.2401 | 0.1154 | 0.0139 | 0.4662 | <0.001 |  |
| Moderate | Indirect | 0.0189 | 0.0033 | 0.0124 | 0.0254 | <0.001 | 12.57 | 0.0309 | 0.0150 | 0.0014 | 0.0604 | <0.001 | 20.73 |
|  | Direct | 0.1303 | 0.0084 | 0.1139 | 0.1467 | <0.001 |  | 0.1112 | 0.0541 | 0.0052 | 0.2172 | <0.001 |  |
|  | Total | 0.1493 | 0.0093 | 0.1310 | 0.1676 | <0.001 |  | 0.1421 | 0.0691 | 0.0066 | 0.2775 | <0.001 |  |
| Severe | Indirect | 0.1128 | 0.0191 | 0.0753 | 0.1503 | <0.001 | 18.37 | 0.0290 | 0.0015 | 0.0261 | 0.0318 | <0.001 | 29.99 |
|  | Direct | 0.4877 | 0.0560 | 0.3780 | 0.5974 | <0.001 |  | 0.0542 | 0.0028 | 0.0488 | 0.0597 | <0.001 |  |
|  | Total | 0.6005 | 0.0707 | 0.4620 | 0.7390 | <0.001 |  | 0.0832 | 0.0042 | 0.0749 | 0.0915 | <0.001 |  |
| Extremely Severe | Indirect | 0.0675 | 0.0164 | 0.0354 | 0.0996 | <0.001 | 29.27 | 0.0400 | 0.0020 | 0.0360 | 0.0439 | <0.001 | 34.62 |
|  | Direct | 0.1585 | 0.0362 | 0.0875 | 0.2295 | <0.001 |  | 0.0582 | 0.0029 | 0.0525 | 0.0639 | <0.001 |  |
|  | Total | 0.2260 | 0.0511 | 0.1259 | 0.3261 | <0.001 |  | 0.0982 | 0.0049 | 0.0885 | 0.1079 | <0.001 |  |
| ACS patients |  |  |  |  |  |  |  |  |  |  |  |  |  |
| Mild | Indirect | 0.0735 | 0.0231 | 0.0283 | 0.1187 | <0.001 | 12.79 | 0.1190 | 0.0182 | 0.0834 | 0.1546 | <0.001 | 11.05 |
|  | Direct | 0.4802 | 0.0510 | 0.3802 | 0.5802 | <0.001 |  | 0.7138 | 0.1089 | 0.5005 | 0.9272 | <0.001 |  |
|  | Total | 0.5537 | 0.0530 | 0.4498 | 0.6576 | <0.001 |  | 0.8329 | 0.1270 | 0.5839 | 1.0818 | <0.001 |  |
| Moderate | Indirect | 0.0491 | 0.0125 | 0.0246 | 0.0735 | <0.001 | 15.45 | 0.0554 | 0.0042 | 0.0471 | 0.0637 | <0.001 | 12.76 |
|  | Direct | 0.0722 | 0.0184 | 0.0362 | 0.1082 | 0.038 |  | 0.3150 | 0.0240 | 0.2680 | 0.3621 | <0.001 |  |
|  | Total | 0.1213 | 0.0309 | 0.0608 | 0.1818 | 0.041 |  | 0.3704 | 0.0282 | 0.3151 | 0.4258 | <0.001 |  |
| Extremely Severe | Indirect | 0.0284 | 0.0058 | 0.0171 | 0.0398 | <0.001 | 29.91 | 0.0609 | 0.0046 | 0.0518 | 0.0701 | <0.001 | 21.90 |
|  | Direct | 0.0429 | 0.0233 | -0.0029 | 0.0886 | 0.158 |  | 0.1933 | 0.0147 | 0.1644 | 0.2222 | <0.001 |  |
|  | Total | 0.0713 | 0.0135 | 0.0449 | 0.0977 | <0.001 |  | 0.2542 | 0.0194 | 0.2162 | 0.2922 | <0.001 |  |

Note: Referenced to HC and adjusted by sex and age. Severe CKD risk cases are missing in the ACS subgroup. A similar reciprocal mediation in the subgroup was also observed, analogous to the overall analysis. NLR, neutrophil-to-lymphocyte ratio; TyG, triglyceride-glucose index; CKD, chronic kidney disease; SE, standard error.
